# The stem cell/cancer stem cell marker ALDH1A3 regulates the expression of the survival factor tissue transglutaminase, in mesenchymal glioma stem cells

**DOI:** 10.18632/oncotarget.16479

**Published:** 2017-03-22

**Authors:** Kelly E. Sullivan, Kathy Rojas, Richard A. Cerione, Ichiro Nakano, Kristin F. Wilson

**Affiliations:** ^1^ Department of Molecular Medicine, Cornell University, Ithaca, NY, USA; ^2^ Department of Chemistry and Chemical Biology, Cornell University, Ithaca, NY, USA; ^3^ Department of Neurosurgery, University of Alabama at Birmingham, Birmingham, AL, USA

**Keywords:** cancer stem cells, tissue transglutaminase, aldehyde dehydrogenase, glioblastoma, retinoic acid

## Abstract

Tissue transglutaminase (tTG), a dual-function enzyme with GTP-binding and acyltransferase activities, has been implicated in the survival and chemotherapy resistance of aggressive cancer cells and cancer stem cells, including glioma stem cells (GSCs). Using a model system comprising two distinct subtypes of GSCs referred to as proneural (PN) and mesenchymal (MES), we find that the phenotypically aggressive and radiation therapy-resistant MES GSCs exclusively express tTG relative to PN GSCs. As such, the self-renewal, proliferation, and survival of these cells was sensitive to treatment with tTG inhibitors, with a benefit being observed when combined with the standard of care for high grade gliomas (i.e. radiation or temozolomide). Efforts to understand the molecular drivers of tTG expression in MES GSCs revealed an unexpected link between tTG and a common marker for stem cells and cancer stem cells, Aldehyde dehydrogenase 1A3 (ALDH1A3). ALDH1A3, as well as other members of the ALDH1 subfamily, can function in cells as a retinaldehyde dehydrogenase to generate retinoic acid (RA) from retinal. We show that the enzymatic activity of ALDH1A3 and its product, RA, are necessary for the observed expression of tTG in MES GSCs. Additionally, the ectopic expression of ALDH1A3 in PN GSCs is sufficient to induce the expression of tTG in these cells, further demonstrating a causal link between ALDH1A3 and tTG. Together, these findings ascribe a novel function for ALDH1A3 in an aggressive GSC phenotype via the up-regulation of tTG, and suggest the potential for a similar role by ALDH1 family members across cancer types.

## INTRODUCTION

Tissue transglutaminase (tTG) is a dual-function GTP-binding protein/crosslinking enzyme which has been previously linked to the development of aggressive cancers. We and others have described roles for tTG in the survival, chemotherapy resistance, migration, and regulation of EGF receptor signaling in a variety of cancer cell types, including glioblastoma [1–7]. Recently, tTG has also been implicated in the survival and proliferation of CD44^+^ glioma stem cells (GSCs), as well as the survival, migration, invasion, and self-renewal of epidermal squamous cell carcinoma stem cells [8–9]. Building on these studies and our previous characterization of tTG in glioblastoma cell lines, we sought to further understand the role of tTG in high grade gliomas (HGGs), specifically in GSCs, as well as how it may be therapeutically targeted, and the mechanism for its up-regulated expression in cancer stem cell (CSC) populations.

To do so, we used GSCs that we previously derived from HGGs as a model system. These GSCs were classified as either mesenchymal (MES) or proneural (PN) based on their gene expression signatures, and they exhibit distinct phenotypes [10]. MES GSCs display a highly aggressive phenotype characterized by an elevated capacity for self-renewal, proliferation, and tumorigenicity in an orthotopic mouse model of HGG, whereas PN GSCs exhibit a much lower rate of proliferation and self-renewal, and generate much less aggressive tumors in mice. Additionally, a number of stem cell/CSC markers were found to be differentially expressed between PN and MES GSCs, with PN GSCs characterized by the expression of CD133, SOX2, and Olig2, whereas CD44 and ALDH1A3 were detected in MES GSCs [10]. Interestingly, we observed that tTG is also expressed specifically in MES GSCs, with no detectable tTG protein levels in PN GSCs. We were thus interested in determining how tTG expression is induced specifically in MES GSCs.

Several mechanisms have been described as contributing to the expression of tTG in a variety of cell types. These include the up-regulation of tTG protein and/or mRNA levels downstream of growth factors and cytokines, such as EGF in breast cancer cells, TGF-β in ovarian cancer and dermal fibroblasts, and IL-6 in hepatoblastoma cells [2, 4, 11–13]. Moreover, the gene encoding tTG, *TGM2*, is a well-known transcriptional target of retinoic acid (RA). The promoter region of *TGM2* contains an RA-response element (RARE), which is bound by a heterodimer comprised of the retinoic acid receptor (RAR) and the retinoid X receptor (RXR) [14–15]. In the absence of RA, the RAR/RXR heterodimer recruits co-repressors that lead to histone deacetylation and the subsequent repression of transcription. However, in the presence of RA, the RAR/RXR heterodimer releases the co-repressor complexes from the *TGM2* promoter, and instead recruits co-activator complexes that promote histone acetylation and gene transcription [16–18].

In exploring whether these mechanisms contribute to tTG expression in MES GSCs, we hypothesized that these highly aggressive cells may exhibit enhanced RA-induced gene transcription downstream of ALDH1A3, a known marker of MES GSCs that has been shown to be important for the proliferation and maintenance of the MES GSC phenotype [10]. Members of the ALDH1 family of proteins function as retinaldehyde dehydrogenases that catalyze the conversion of retinal to RA; thus, these enzymes likely play an important role in the regulation of gene expression, and when de-regulated, may help drive the CSC phenotype [16, 19–20]. In particular, ALDH1A1 and ALDH1A3 have been found to be markers of CSCs of various tissue origins, including tumors of the brain, head and neck, breast, liver, lung, ovaries, pancreas, prostate, colon, bladder, and skin, as well as leukemia [10, 19, 21–31]. However, while a growing body of evidence suggests that ALDH1 family proteins are critical for maintaining the stem cell-like properties of CSCs, very little is known regarding the mechanism by which these enzymes support self-renewal and tumor initiation. Furthermore, ALDH1^+^ CSCs are not readily susceptible to therapeutic intervention, exhibiting resistance to most standard therapies, including chemotherapy and radiation [32–34]. Given the potentially significant role of ALDH1 family enzymes in tumor initiation, resistance, and recurrence, a deeper understanding of these enzymes in CSCs is warranted. As such, we chose to investigate whether tTG expression may be driven by ALDH1A3-induced RA signaling in MES GSCs.

Here, we show that the up-regulated expression of tTG in MES GSCs offers a unique strategy for the therapeutic targeting of these highly aggressive tumor-initiating cells. We go on to demonstrate that combining a tTG inhibitor with either radiation or temozolomide (TMZ) not only impairs self-renewal and proliferation in MES GSCs, but also potently induces cell death. Interestingly, we found that tTG is indeed induced downstream of RA and ALDH1A3 in MES GSCs, and its expression can be up-regulated in PN GSCs by the introduction of RA or ALDH1A3. This mechanism for tTG expression appears to be conserved in other cancer cell types, as demonstrated by the comparison of ALDH1^high^ and ALDH1^low^ cancer cell populations. Taken together, our results suggest that tTG may represent a novel therapeutic target for aggressive GSCs and other ALDH1+ cancer cells, as well as provide insight into the contributions of ALDH1A3 to the CSC phenotype.

## RESULTS

### tTG is differentially expressed between MES and PN GSCs and provides a therapeutic target for the elimination of MES GSCs

Earlier work identified two mutually exclusive subtypes of GSCs present in HGGs, classified as proneural (PN) or mesenchymal (MES) based on their gene expression signatures. One marker that distinguishes PN versus MES GSCs is the CSC protein CD44, which is present in the MES subtype but not in the PN subtype [10]. It has been reported that the expression of tissue transglutaminase (tTG) is associated with the expression of CD44 in ovarian cancer as well as in glioma-initiating cells, and that the genetic silencing or pharmaceutical inhibition of tTG in the latter is sufficient to impair cell proliferation and induce apoptosis in these cells [8, 35]. Thus, it was of interest to determine whether the expression of tTG could distinguish the PN and MES subtypes of GSCs, and thereby potentially serve as a pharmaceutical target of MES GSCs.

**Figure 1:**
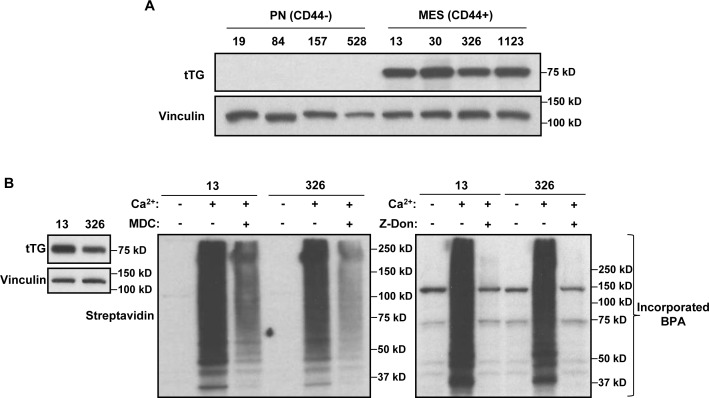
MES GSCs exclusively express tTG relative to PN GSCs and are sensitive to the effects of tTG inhibitors **A**. Whole cell lysates from PN and MES GSCs were immunoblotted with tTG, and Vinculin antibodies. **B**. Whole cell lysates collected from MES GSC cell lines 13 and 326 were immunoblotted with tTG and Vinculin antibodies (left panel), or incubated with or without MDC (middle panel) and Z-Don (right panel). tTG transamidation activity was read-out by the incorporation of a biotinylated-pentylamine onto cell lysates, and detected with a Streptavidin antibody.

**Figure 2:**
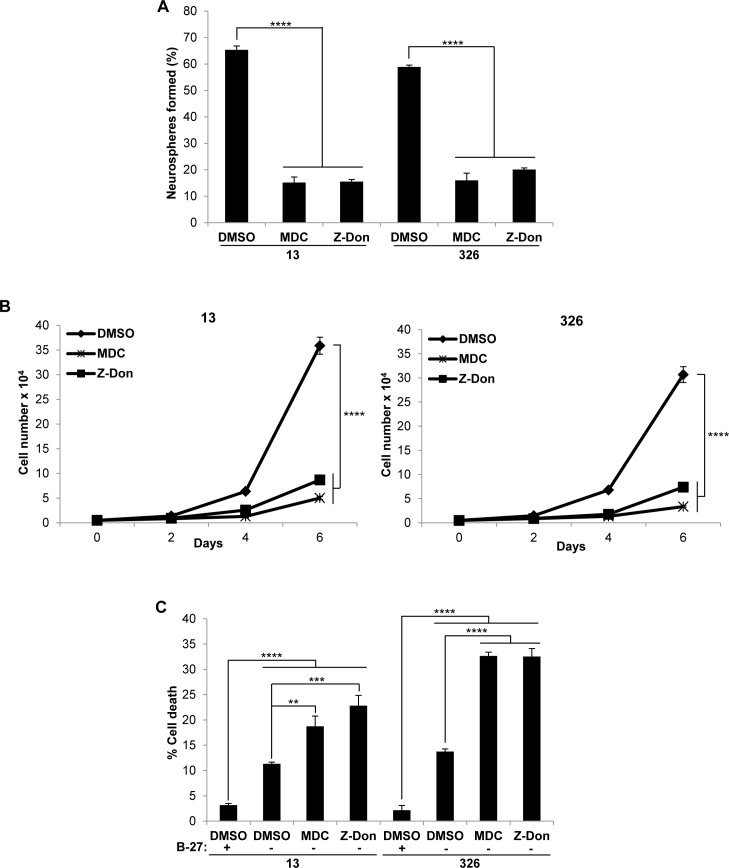
Pharmaceutical inhibitors of tTG impact the self-renewal, proliferation and survival of MES GSC **A**., **B**. MES GSC cell lines 13 and 326 were dissociated into single cells and seeded at 5 × 10^3^ cells/well in 12-well plates. **A**. Neurospheres were counted after 72 hours. Each experiment was performed in triplicate, and the results were averaged and graphed. p values are represented as follows: ****, *p* < 0.0001. **B**. The cells were counted at the indicated time points to determine cell proliferation. Each experiment was performed in triplicate, and the results were averaged and graphed. p values are represented as follows: ****, *p*< 0.0001. **C**., tTG inhibitors induce cell death following nutrient deprivation. MES GSC cell lines 13 and 326 were dissociated into single cells, and seeded at 2 × 10^4^ cells/well in 12-well plates in either GSC medium or DMEM/F12 with the indicated compounds. The cells were collected after 48 hours, stained with Trypan Blue Solution, and the viable and dead cells were counted. Each experiment was performed in triplicate, and the results were averaged and graphed. p values are represented as follows: **, *p* < 0.01; ***, *p* < 0.001; ****, *p* < 0.0001.

As a first step, we screened a panel of 4 PN and 4 MES GSC lines for the presence of tTG by Western blotting. Figure 1A shows that each of the 4 MES GSC lines robustly expresses tTG protein, while it was undetectable in each of the 4 PN GSC lines. We then went on to confirm that the tTG-expressing MES GSC lines were susceptible to treatment with two commonly used tTG inhibitors, MDC and Z-Don, with respect to their self-renewal, proliferation and survival. MDC functions as a competitive inhibitor of tTG by acting as an alternative amine donor for the transamidase activity of tTG, whereas Z-Don binds irreversibly to the transamidation active site cysteine, inhibiting enzymatic (acyltransferase) activity (for example, see Figure 1B) [36–37]. We found that disabling the acyltransferase activity of tTG with each of these inhibitors blocked the ability of the MES GSC cell lines 13 and 326 to form neurospheres, thereby preventing the cells from undergoing self-renewal (Figure 2A). Similarly, the proliferation of the MES GSCs was significantly reduced when treated with MDC and Z-Don (Figure 2B). Moreover, blocking tTG activity impacted the ability of these cells to survive when stressed. This was assessed by starving the MES GSC lines 13 and 326 of essential nutrients (i.e. through the removal of heparin and B-27), in the presence or absence of MDC and Z-Don. Specifically, we found that while nutrient starvation slightly induced cell death, a significant enhancement in apoptosis was observed for cells exposed to the tTG inhibitors (Figure 2C). Taken together, these findings support the idea that tTG inhibitors have the potential for providing therapeutic benefit in HGGs that are characterized by the presence of MES GSCs.

### Combining tTG inhibitors with chemotherapeutic agents and radiation induces cell death

GSCs exhibit tumor-initiating properties as well as enhanced resistance to chemotherapeutic drugs and radiotherapy compared to non-GSCs, and as such, they are thought to be the primary drivers of tumor recurrence [32, 34, 38–40]. Therefore, therapies designed to target these cells may provide additional benefit over traditional methods, especially for the treatment of HGG where tumor recurrence is the typical outcome. We thus examined the benefits of combining tTG inhibitors with the current standard of care for HGG, namely radiation and TMZ [34]. To perform these experiments, we wanted to use sub-optimal levels of each therapy in order to visualize any additive or synergistic affects that might occur when they were then used in combination. We first determined the IC_50_ values for Z-Don in the MES GSC 13 and 326 cell lines using dose curve proliferation assays, and compared them to the glioblastoma cell line T98G (a cell line which expresses very little tTG, and is insensitive to treatment with Z-Don) (Supplemental Figure 1A-D). IC_50_ values were similarly determined for radiation and TMZ in the MES GSC 13 and 326 cell lines (Supplemental Figure 1E, F).

**Figure 3:**
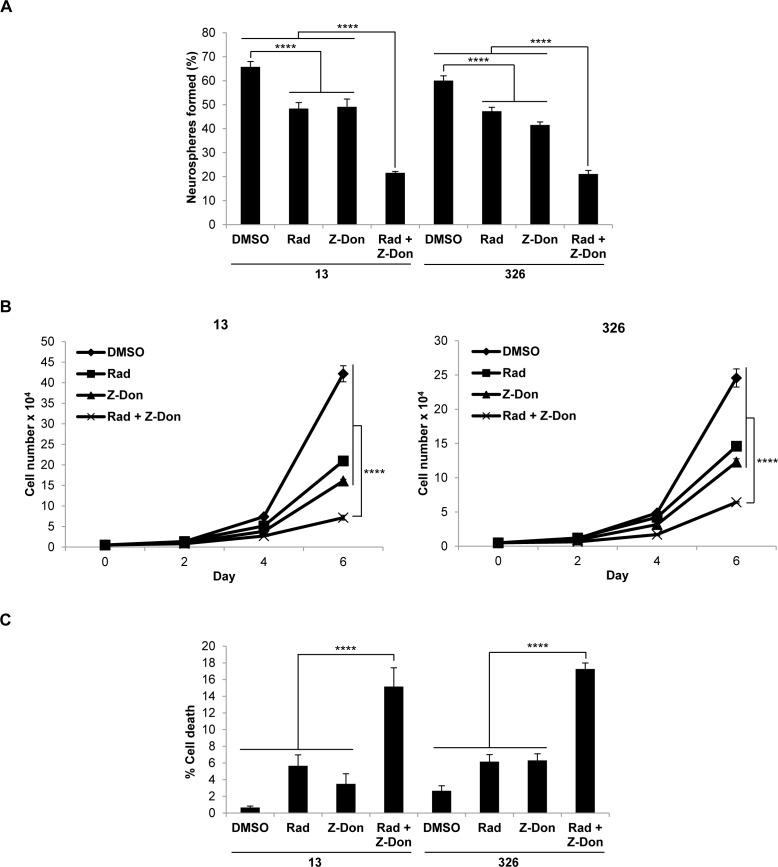
Combination therapy including the tTG inhibitor Z-Don and radiation inhibits MES GSC self-renewal and proliferation, and induces cell death A-C. MES GSC cell lines 13 and 326 were dissociated into single cells, seeded at 5 × 10^3^ cells/well in 12-well plates with or without Z-Don, and radiated after 2-4 hours. **A**. Neurosphere formation was counted after 72 hours. Each experiment was performed in triplicate, and the results were averaged and graphed. p values are represented as follows: ****, *p* < 0.0001. **B**. The cells were counted at the indicated time points to determine cell proliferation. Each experiment was performed in triplicate, and the results were averaged and graphed. *p* values are represented as follows: ****, *p* < 0.0001. **C**. The cells were collected after six days and stained with Trypan Blue Solution, and the viable and dead cells were counted. Each experiment was performed in triplicate, and the results were averaged and graphed. *p* values are represented as follows: ****, *p* < 0.0001.

**Figure 4:**
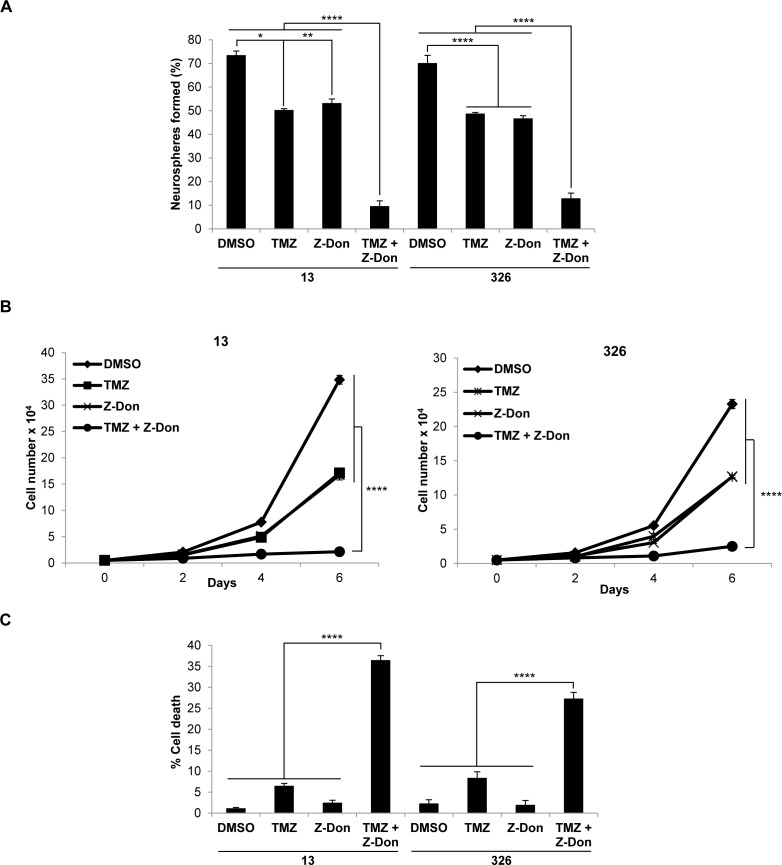
Combination therapy including the tTG inhibitor Z-Don and temozolomide inhibits MES GSC self-renewal and proliferation, and induces cell death **A**.-**C**. MES GSC cell lines 13 and 326 were dissociated into single cells, and seeded at 5 × 10^3^ cells/well in 12-well plates with or without the indicated compounds. **A**. Neurosphere formation was counted after 72 hours. Each experiment was performed in triplicate, and the results were averaged and graphed. *p* values are represented as follows: *, *p* < 0.05; **, *p* < 0.01; ****, *p* < 0.0001. **B**. The cells were counted at the indicated time points to determine cell proliferation. Each experiment was performed in triplicate, and the results were averaged and graphed. p values are represented as follows: ****, *p* < 0.0001. **C**. The cells were collected after six days and stained with Trypan Blue Solution, and the viable and dead cells were counted. Each experiment was performed in triplicate, and the results were averaged and graphed. *p* values are represented as follows: ****, *p* < 0.0001.

We then radiated MES GSC cell lines 13 and 326, and treated them with or without Z-Don, at their IC_50_ doses for both radiation and Z-Don. A decrease in neurosphere formation and proliferation, as well as an increase in cell death, was observed with the combination treatment compared to either radiation or Z-Don treatment alone (Figures 3A-3C). Likewise, when we treated MES GSCs with a combination of TMZ and Z-Don at their IC_50_ doses, we again observed an inhibition of self-renewal and proliferation (Figures 4A and 4B). Strikingly, cell viability assays showed that while sub-lethal doses of TMZ and Z-Don individually had little impact on MES GSC survival, the combination therapy caused a synthetic lethality (Figure 4C).

### Determining the glioma stem cell drivers of tTG expression

**Figure 5:**
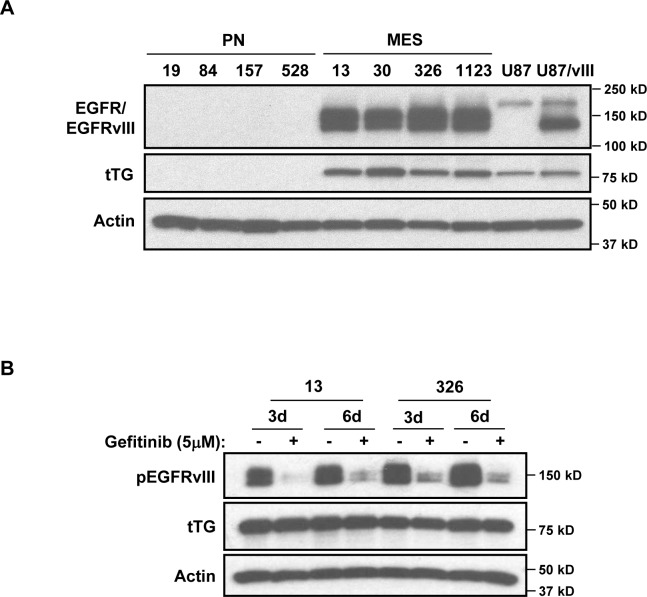
Determining potential upstream regulators of tTG expression in MES GSCs **A**. A lower molecular weight form of EGFR is expressed exclusively in MES GSCs. Whole cell lysates from PN and MES GSCs were immunoblotted with EGFR, tTG, and Actin antibodies. **B**. EGFR inhibition has no effect on tTG expression. MES 13 and 326 cells were treated with 5 μM Gefitinib for either 3 or 6 days, and the effects on tTG expression were determined by Western blotting using antibodies against tTG, phospho-EGFR and actin.

Together, these data further reinforce various findings that suggest tTG may offer a potentially new therapeutic target against aggressive cancers, including HGGs. However, from a mechanistic perspective, we wanted to understand what drives the specific expression of tTG in the highly aggressive MES GSCs, in contrast to the lack of expression of tTG in the less aggressive PN GSCs. Such knowledge could provide valuable insights into the types of cancers that would be most susceptible to the use of tTG inhibitors. To this end, we examined what other proteins are differentially expressed between MES and PN GSCs, and thereby might impact tTG expression. We have reported previously that in certain types of cancer cells, tTG expression can be up-regulated through EGFR-dependent signaling, and so we investigated whether there was an apparent difference in EGFR expression status between MES and PN GSCs [2, 4]. In fact, we observed the strong expression of a lower molecular weight form of the EGFR in MES GSCs, whose mobility was consistent with that of the truncated EGFR oncogenic mutant, EGFRvIII (Figure 5A, compare the EGFR present in the MES GSC cell lines to the endogenous EGFR expressed in human glioblastoma U87 cells, or with the ectopic expression of EGFRvIII in U87 cells). This was in sharp contrast to the PN GSC lines where we detected little or no expression of the wild-type EGFR or the EGFRvIII. We then tested whether an EGFR inhibitor, Gefitinib, affected the expression of tTG in MES GSCs. Specifically, we challenged the MES GSC cell lines 13 and 326 with Gefitinib, and then assessed tTG levels after 3 or 6 days of treatment (Figure 5B). Although the phosphorylation of the EGFRvIII was reduced under these conditions, as was cell proliferation (data not shown), tTG levels were largely unaffected.

### The generation of retinoic acid by ALDH1A3 induces the expression of tTG

**Figure 6:**
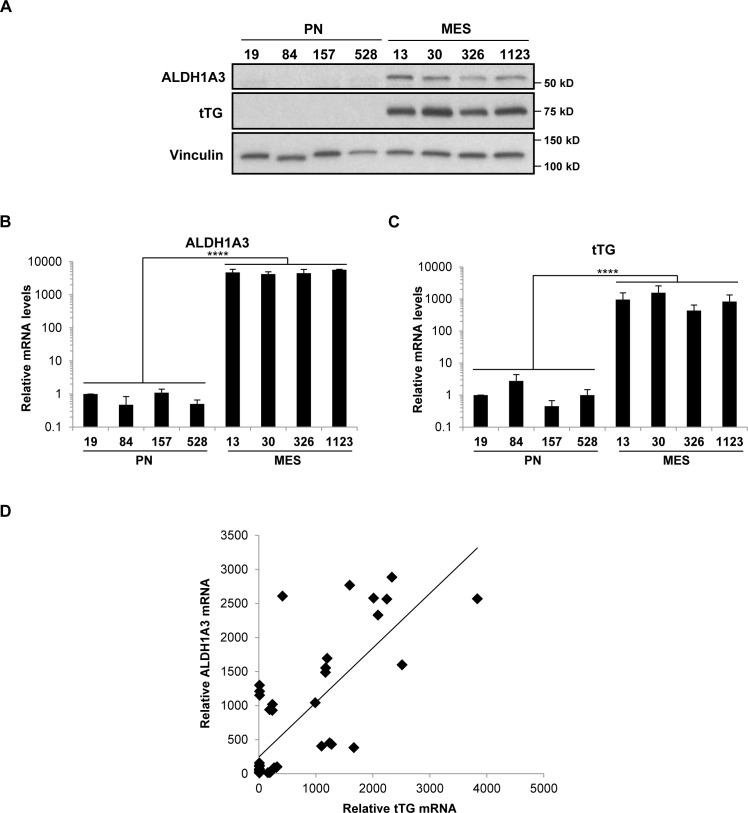
ALDH1A3 and tTG are expressed exclusively in MES GSCs **A**. Whole cell lysates from PN and MES GSCs were immunoblotted with ALDH1A3, tTG, and Vinculin antibodies. **B**., **C**. RNA was isolated from PN and MES GSCs, and cDNA was generated as described in “Materials and Methods.” qPCR was then performed with primer sets that amplify ALDH1A3 and tTG transcripts, and the results of three independent experiments were averaged and plotted with the PN GSC 19 cell line normalized to one. p values are represented as follows: ****, *p* < 0.0001. **D**. ALDH1A3 and tTG mRNA levels are correlated in GSC, GBM, and astrocyte cell lines.

As signaling through the EGFRvIII did not appear to contribute to tTG expression in MES GSCs, we examined the potential role of another known driver of tTG expression, retinoic acid (RA). Both PN and MES GSC lines are cultured in the presence of media containing Vitamin A, the precursor of RA. Interestingly, MES GSCs are known to highly express ALDH1A3, a member of the ALDH1 family of retinaldehyde dehydrogenases that converts retinal to RA. Not only is ALDH1A3 highly expressed, but it is also the only retinaldehyde dehydrogenase expressed in MES GSCs, underscoring the importance of ALDH1A3 for RA-dependent gene regulation in these cells [10]. We confirmed, by Western blotting and qRT-PCR, that ALDH1A3 is expressed at high levels in MES GSCs, whereas it is nearly undetectable in PN GSCs (Figures 6A and 6B), as was previously reported [10]. Furthermore, the expression of tTG appears to be correlated with ALDH1A3 expression in both the MES GSC cell lines 13 and 326 (compare Figure 6C to Figure 6B), as well as a panel of patient-derived GSC cell lines, glioblastoma cell lines, and astrocytes (Figure 6D).

**Figure 7:**
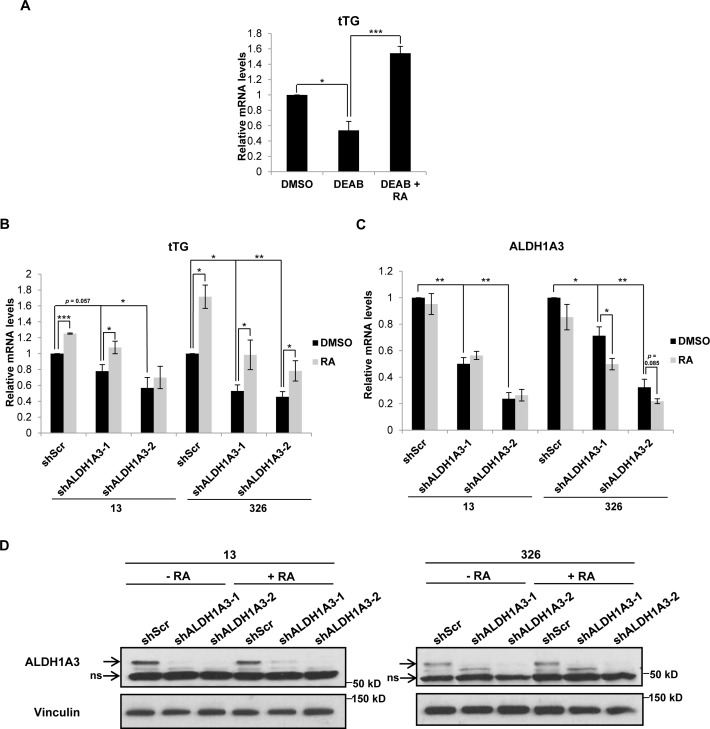
ALDH1A3 is necessary for tTG expression in MES GSCs **A**. MES GSC cell line 326 was treated with the indicated compounds for seven days, followed by RNA isolation and qRT-PCR analysis of tTG transcript levels. The results of independent experiments (n ≥ 3) were averaged and plotted. p values are represented as follows: *, *p* < 0.05; ***, *p* < 0.001. **B**., **C**. MES GSC cell lines 13 and 326 were infected with a control lentivirus or lentiviruses containing two distinct ALDH1A3 shRNAs. The cells were split after 24 hours, and treated with either DMSO or RA and selected with puromycin for six days. The cells were then collected for RNA isolation and qRT-PCR analysis of tTG (B) and ALDH1A3 (C) expression. The results of three independent experiments were averaged and plotted. p values are represented as follows: *, *p* < 0.05; **, *p* < 0.01; ***, *p* < 0.001. **D**. A population of the MES GSC 13 and 326 cells collected in B and C above were used to make whole cell lysates, which were immunoblotted with ALDH1A3 and Vinculin antibodies.

We examined whether tTG expression in MES GSCs is dependent on the ability of ALDH1A3 to generate RA and found that upon treating the MES GSC cell line 326 for seven days with DEAB, an inhibitor of the enzymatic activity of ALDH1 family proteins, there was a marked reduction in tTG mRNA levels that could be rescued upon treatment with RA (Figure 7A). Knocking down ALDH1A3 expression in MES GSC cell lines 13 and 326 also gave rise to a significant reduction in tTG mRNA levels, which was again rescued by the addition of RA (Figure 7B). ALDH1A3 expression was down-regulated in response to RA in the MES GSC 326 cell line, in agreement with previous reports describing the negative regulation of ALDH1A3 transcription by RA (Figure 7C) [19]. Taken together, these data show that the production of RA by ALDH1A3 induces the expression of tTG in MES GSCs.

**Figure 8:**
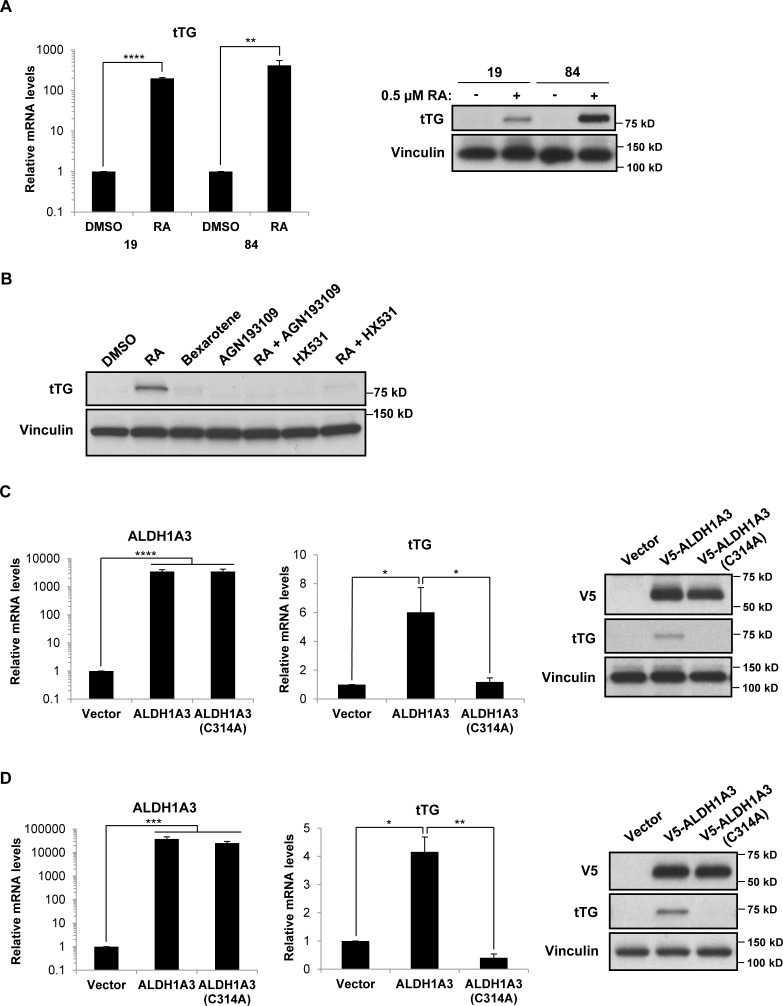
Retinoic acid and ALDH1A3 are sufficient to induce the expression of tTG in PN GSCs **A**. PN GSC cell lines 19 and 84 were treated with 0.5 μM RA for 72 hours, then collected for RNA isolation and qRT-PCR analysis of tTG expression (left panel). The results of three independent experiments were averaged and plotted. p values are represented as follows: **, *p* < 0.01; ****, *p* < 0.0001. Whole cell lysates were collected in parallel, and immunoblotted with tTG and Vinculin antibodies (right panel). **B**. PN GSC 19 cells were treated for 3 days with 0.5 μM RA, 10 μM bexarotene, 1 μM AGN193109, 2 μM HX531, or the indicated combinations of these reagents. Whole cell lysates were collected in parallel, and immunoblotted with tTG and Vinculin antibodies. **C**., **D**. PN GSC cell lines 19 (C) and 84 (D) were infected with a control lentivirus or lentiviruses containing either a wild-type or catalytically-inactive form of ALDH1A3. The cells were split 24 hours later and selected with puromycin for six days, followed by RNA isolation and qRT-PCR analysis of ALDH1A3 and tTG expression (left and middle panels). The results of independent experiments (*n* ≥ 3) were averaged and graphed. p values are represented as follows: *, *p* < 0.05; **, *p* < 0.01; ***, *p* < 0.001, ****, *p* < 0.0001. Whole cell lysates were collected in parallel, and immunoblotted with V5, tTG, and Vinculin antibodies (right panels).

We then set out to determine whether ALDH1A3 was sufficient to induce tTG expression in GSCs that do not normally express these proteins, specifically, by taking advantage of the ALDH1A3^−^/tTG^−^ PN GSC cell lines 19 and 84. We first treated these cells with RA for three days, and then determined the levels of tTG expression by qRT-PCR and Western blotting. Figure 8A shows that tTG transcript (left panel) and protein levels (right panel) were in fact induced upon treatment of PN GSCs with RA, although the PN GSC 84 cell line exhibited a more robust expression of tTG compared to the PN GSC 19 cell line. To determine whether this RA-induced tTG expression was dependent on RAR/RXR-activated transcription, we treated the PN GSC 19 cells with RAR and RXR agonists and antagonists, alone and in combination, and analyzed tTG expression via Western blot. We again observed that RA is able to induce tTG expression, but an RXR agonist, bexarotene, does not result in any appreciable tTG protein levels. Furthermore, co-treating these cells with RA and an RAR antagonist (AGN193109) or an RXR antagonist (HX531) abolishes the effects of RA on tTG expression (Figure 8B). These results are consistent with previous studies of the transcriptional regulation of RAREs, which demonstrate that the RAR and RXR are functionally active as heterodimers, and that RA is required for their activation [16–18]. To more directly examine the role of ALDH1A3 in regulating tTG expression levels, we generated a V5-tagged wild-type ALDH1A3 expression construct, as well as a V5-tagged catalytically inactive mutant of the enzyme (ALDH1A3(C314A)). This mutation targets a strictly conserved cysteine within the enzyme active site of aldehyde dehydrogenase family members, and has been shown to render the enzyme incapable of producing RA [41–43]. These constructs, or an empty vector as a control, were introduced into the PN GSC cell lines 19 (Figure 8C) and 84 (Figure 8D) using a lentiviral system, and cells stably expressing each construct were generated. Seven days post-infection, we observed a significant increase in the expression of each ALDH1A3 construct (Figures 8C and 8D, left and right panels). Additionally, we found that the ectopic expression of wild-type ALDH1A3 induced tTG expression, whereas the catalytically inactive ALDH1A3 was ineffective, as read out by qRT-PCR (Figures 8C and 8D, middle panels) and Western blotting (Figures 8C and 8D, right panels). Thus, ALDH1A3 is sufficient to induce tTG expression in PN GSCs, and this induction is dependent on the production of RA through ALDH1A3 catalysis.

**Figure 9:**
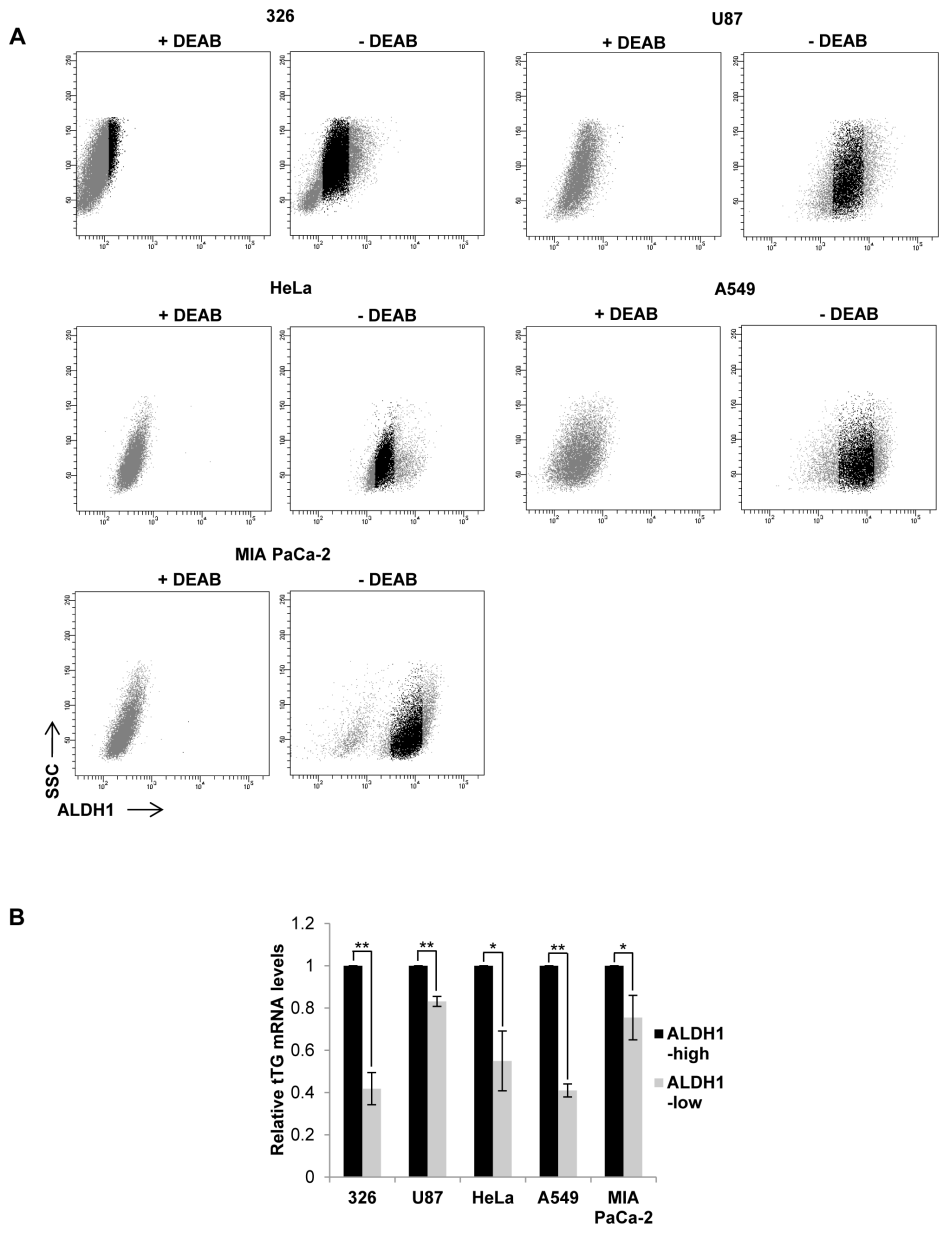
tTG expression is correlated with ALDH1 activity **A**. 5 different cancer cell lines were collected and stained using the ALDEFLUOR kit as described in “Materials and Methods.” The untreated cells (“- DEAB”) with the top 15% and bottom 15% ALDH1 activity (shown in gray) were gated as indicated and collected. **B**. RNA was isolated from the cells collected in A for qRT-PCR analysis of tTG expression. The results of independent experiments (*n* ≥ 3) were averaged and graphed. p values are represented as follows: *, *p* < 0.05; **, *p* < 0.01; ***, *p* < 0.001.

Based on these results, we next asked whether ALDH1 isozymes induce tTG expression in other cancer cell types aside from MES GSCs. We treated several cancer cell lines with or without the ALDH1 inhibitor DEAB, and then performed flow cytometry analysis to identify ALDH1^high^ and ALDH1^low^ populations. The untreated cells were then sorted and collected, followed by qRT-PCR analysis for tTG expression (Figures 9A and 9B). As expected, we observed that in the MES GSC 326 cell line, tTG transcript levels are significantly lower in the ALDH1^low^ cells compared with the ALDH1^high^ population, exhibiting a 50% decrease in tTG expression. Interestingly, we found that this relationship between ALDH1 activity and tTG expression is conserved in the more differentiated glioblastoma cell line U87, as well as in HeLa cervical carcinoma cells, A549 lung carcinoma cells, and MIA PaCa-2 pancreatic carcinoma cells. These data demonstrate that the induction of tTG expression by ALDH1A3 in MES GSCs is maintained in other cell types expressing ALDH1 isozymes, thus raising the intriguing possibility that tTG may provide a sensitive marker and therapeutic target for MES GSCs and HGG, as well as in other cancer cells expressing ALDH1 family enzymes.

## DISCUSSION

**Figure 10:**
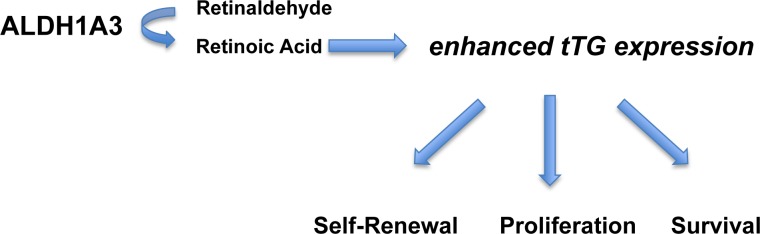
ALDH1A3 promotes stem cell-like properties by enhancing the expression of tTG ALDH1A3 converts retinaldehyde to retinoic acid, which induces the expression of tTG. tTG contributes to the aggressive phenotype of MES GSCs by promoting their self-renewal, proliferation, and survival.

In previous work, we demonstrated that GSCs could be subtyped based on their gene expression profiles into two classes, proneural (PN) and mesenchymal (MES), with the CD44^+^ MES GSCs showing a markedly more aggressive phenotype and radio-resistance relative to PN GSCs [10]. By understanding the unique molecular drivers responsible for the MES phenotype, therapeutic strategies that effectively target these GSCs can be developed. Thus, we set out to determine whether tTG might serve as a marker protein that could distinguish these two distinct GSC subtypes, based on reports that tTG correlates with the expression of CD44 in GSCs and is necessary for their proliferation [8]. We found that the MES GSCs, but not the PN GSCs, robustly express tTG, and that their abilities to self-renew, proliferate, and survive were each susceptible to tTG pharmacological inhibitors. Additionally, we went on to attribute the specificity of tTG expression in MES GSCs to the stem cell and CSC marker, ALDH1A3 (see below and Figure 10).

Our laboratory and others have previously demonstrated key roles for tTG in various aspects of the cancer cell phenotype. These functions include the up-regulation of EGFR levels in glioblastoma through the binding of tTG to c-Cbl, the migration of HeLa cervical carcinoma cells and MDA-MB231 breast cancer cells through an interaction with Hsp70, and the docking of cancer cell-derived microvesicles onto recipient cells through the tTG-mediated cross-linking of fibronectin [5–7]. Not only has tTG been found to play a number of different roles in cellular transformation, but its expression is often significantly elevated, especially in aggressive forms of cancer, and in CSCs derived from these tumors. tTG expression has been shown to contribute to the stemness and survival of CD44^+^ GSCs, epidermal CSCs, CD44^+^/CD117^+^ ovarian CSCs, and CD44^+^/CD24^−^ breast CSCs [8, 9, 11, 44]. Thus, we anticipated a similar role for tTG in the highly aggressive phenotype of MES GSCs, and expected these cells to be sensitive to tTG inhibitors. What we did not anticipate, however, was the striking efficacy of these inhibitors when used in combination with the standard of care treatments. In particular, the use of TMZ and Z-Don in combination potently induced cell death at concentrations which had little effect on the cells when used alone. These results underscore the potential for tTG inhibitors in the clinic, and highlight the need for the development of clinically relevant small molecules that target tTG.

While it is well documented that tTG is highly expressed in many cancer types and CSCs, there is still a good deal to learn about the mechanisms by which this important survival factor is being up-regulated. The correlation that exists between CD44 and tTG expression across a number of CSC types (as noted above) is intriguing, yet it is not obvious how the CD44 cell-surface glycoprotein which is involved in cell-cell interactions, adhesion, and migration, might causally influence tTG expression. We have shown in different breast cancer cell lines (such as SKBR3, MDA-MB468, and BT20) that EGF is sufficient to call up tTG expression and activation [2, 4]. Given the robust expression of a lower molecular weight form of EGFR present in MES GSCs but not PN GSCs, we initially examined the potential role of EGFR signaling in the regulation of tTG expression in MES GSCS. When it was clear that there did not appear to be a functional connection between EGFR and tTG expression in these cells, we next considered RA as a possible regulator of tTG expression in MES GSCs, as RA has been shown to induce tTG expression in different cell types [1, 2, 45]. ALDH1A3, a stem cell and CSC marker which functions as a retinaldehyde dehydrogenase in cells to produce RA, is exclusively expressed in MES GSCs, but not in PN GSCs.

Although numerous studies have classified both ALDH1A1 and ALDH1A3 as markers of CSCs derived from several distinct tumor types, the functional roles of these enzymes in CSCs have not been well-defined [19]. Limited studies by us and others have suggested that ALDH1A3 supports stemness in GSCs by promoting glycolysis/gluconeogenesis, and is associated with higher Stat3 signaling in ALDH1^+^ CSCs derived from non-small cell lung cancer [10, 24]. A report examining melanoma CSCs described a number of genes that appear to be regulated by ALDH1A1 and/or ALDH1A3, including CDC42, a gene containing RAREs [30]. Here, we have investigated the role of ALDH1A3 in cancer progression using ALDH1A3^+^ and ALDH1A3^−^ GSCs as a model system. We show that ALDH1A3 regulates the expression of the RARE-containing gene, *TGM2*, in MES GSCs, through its enzymatic conversion of retinaldehyde to RA. Using ALDH1A3 knockdowns and an inhibitor of ALDH1 enzymatic activity, we have found that ALDH1A3 mediates the transcriptional regulation of *TGM2* in MES GSCs. Moreover, the over-expression of ALDH1A3 in PN GSCs is sufficient to induce tTG expression, whereas a catalytically inactive form of this enzyme (ALDH1A3(C314A)) is ineffective. We think it is likely that the RA generated by ALDH1A3 binds to RARs bound to RAREs in the promoter region of *TGM2*, although we cannot, in this study, discount the possibility that RA is regulating the transcription of *TGM2* by an indirect mechanism. Finally, given that ALDH1 family isozymes function as retinaldehyde dehydrogenases, this mechanism for the induction of tTG expression, as well as that of several other RA-regulated genes, may potentially be broadly expanded to include several other types of ALDH1^+^ CSCs and cancer cells [16, 19, 20].

Interestingly, the cellular concentration of RA appears to have a strong impact on the outcome of RA-induced gene expression. RA signaling plays a critical role during development, especially in the differentiation of stem cells into neural progenitors and neurons [46, 47]. *In vitro* experiments aimed at inducing the differentiation of embryonic stem cells into neural progenitor cells typically involve RA concentrations ranging from 5 μM to 5 mM [48]. However, in the case of MES GSCs, the endogenous RA concentration is likely much lower so as to promote the stemness of these cells, rather than inducing their differentiation. Indeed, our unpublished observations have suggested that a relatively low concentration of RA (0.5 μM) induces MES GSCs to become adherent rather than grow as neurospheres in suspension, suggesting that they are transitioning toward a more differentiated state. In treating PN GSCs with 0.5 μM RA, or when over-expressing the wild-type ALDH1A3, we have noted that some of these cells also begin to attach to tissue culture flasks, as well as exhibit slower growth kinetics relative to their untreated counterparts (data not shown). These results suggest that ALDH1A3 and RA alone cannot induce the highly aggressive phenotype of MES GSCs, and likely work together with other tumor promoters or oncogenes (e.g. the EGFRvIII) in MES GSCs to promote their rapid proliferation and tumorigenicity.

The observed sensitivity of PN and MES GSCs to low levels of RA suggests that the concentration of this compound must be carefully regulated in order to maintain the CSC phenotype. In part, this regulation is accomplished through a negative feedback loop that down-regulates the expression of ALDH1 isozymes. Thus, rather than being induced by RA, as are genes containing RAREs, the transcription of ALDH1 genes is instead inhibited by RA, leading to an eventual reduction in the RA concentration [19]. This feedback loop can be observed in Figure 7C, particularly in the MES GSC 326 cell line, in which ALDH1A3 transcript levels are decreased following treatment with RA. Therefore, it appears that nanomolar levels of RA are capable of promoting the CSC phenotype in MES GSCs, whereas higher concentrations of RA seem to induce the differentiation of these cells. Indeed, RA therapy is commonly used for the treatment of acute promyelocytic leukemia, leading to the differentiation of leukemic cells and often resulting in a complete remission [49–51]. These results indicate that proteins in the RA signaling pathway, including ALDH1 isozymes and RAR/RXR heterodimers, have the potential to be further exploited in the treatment of various types of cancer.

In exploring the regulation of gene expression by ALDH1A3 in GSCs, we also sought to understand a potential role for tTG in these cells. tTG has been reported to play an important part in the differentiation of normal stem and progenitor cells during development, such as the differentiation of neural progenitors into neurons and osteoblasts into osteocytes [14, 45, 52]. Notably, ALDH1 proteins are also expressed in many normal stem and progenitor cells, including neural stem cells and hematopoietic stem cells [53]. Thus, the mechanism of tTG induction by ALDH1A3 that we have observed in MES GSCs may be a reflection of a normal stem cell process. However, critical differences in the gene expression profiles of MES GSCs versus normal stem cells likely determine the extent to which tTG promotes differentiation versus tumor progression. In the context of MES GSCs, the high degree of growth factor signaling (e.g. through the EGFRvIII), in combination with other stem cell factors, may overwhelm any differentiation-promoting effects of tTG.

In conclusion, this work further identifies tTG as a potent therapeutic target in HGGs, with the novel identification of tTG as a component of the ALDH1A3-induced expression profile. By taking advantage of our model system involving two distinct (both phenotypically and genotypically) GSC subtypes, we were able to demonstrate that ALDH1A3 is not only necessary for the expression of tTG in MES GSCs, but that it is also sufficient (as well as RA) to induce tTG in PN GSCs when ectopically expressed. These findings provide a mechanism to explain the transcriptional regulation of tTG in MES GSCs, and also provide insight as to the functional significance of common stem cell/CSC markers involving the ALDH1 family.

## MATERIALS AND METHODS

### Cell culture

GSCs were cultured as described previously [10]. Briefly, the cells were maintained in DMEM/F12 supplemented with B-27 (2%), heparin (5 μg/mL), glutamine (4.5 mM), penicillin-streptomycin (100 U/mL), basic FGF (bFGF) (20 ng/mL), and EGF (20 ng/mL). Growth factors (bFGF and EGF) were added every 3-4 days. MES GSCs were dissociated into single cells via gentle pipetting, and PN GSCs were dissociated with TrypLE Express Enzyme and gentle pipetting. Where indicated, MES and PN GSCs were cultured in 0.5 μM RA, PN GSCs were cultured in 10 μM bexarotene, 2 μM HX531, and 1 μM AGN193109, and MES GSCs were cultured in 100 μM diethylaminobenzaldehyde (DEAB). Unless otherwise noted, the MES GSC 13 cell line was treated with 50 μM monodansylcadaverine (MDC) and 40 μM Z-DON-Val-Pro-Leu-OMe (Z-Don), and the MES GSC 326 cell line was treated with 60 μM MDC and 30 μM Z-Don. T98G, HEK293T, and A549 cells were maintained in DMEM containing 10% FBS; U87 and HeLa cells were cultured in RPMI containing 10% FBS; and MIA PaCa-2 cells were maintained in DMEM supplemented with 10% FBS and 2.5% HS. All cells were incubated in a humidified atmosphere with 5% CO_2_ at 37°C.

### Reagents and antibodies

DMEM/F12, DMEM, RPMI, TrypLE Express, trypsin, FBS, HS, Trypan Blue Solution, and puromycin were purchased from Gibco; B-27, penicillin-streptomycin, EGF, and Lipofectamine were from Invitrogen; heparin, glutamine, DEAB, RA, MDC, dimethyl sulfoxide (DMSO), TMZ, bexarotene, and polybrene (hexadimethrine bromide) were from Sigma; bFGF was from Peprotech; polyethylenimine (PEI) was from Polysciences, Inc.; biotinylated pentylamine (BPA) was from Pierce; and Z-Don was from Zedira. HX531 was from Tocris Bioscience. Gefitinib was from Selleck Chemicals. AGN193109 was from Santa Cruz Biotechnology. The ALDEFLUOR kit was from STEMCELL Technologies; QIAshredder and RNeasy Mini Kit were from Qiagen; Superscript III First-Strand Synthesis System was from Invitrogen; and iTaq Universal SYBR Green Supermix was from Bio-Rad. Primary antibodies used in this study were anti-ALDH1A3 rabbit pAb (abcam, ab129815), anti-Transglutaminase II Ab-3 mouse mAb (Cocktail) (Neomarkers, MS-300-P), anti-Vinculin mouse mAb (Sigma, V9131), anti-Actin pan Ab-5 mouse mAb (Thermo Fisher Scientific, MS-1295), anti-EGF Receptor rabbit mAb (Cell Signaling, 4267), anti-Phospho-EGF Receptor (Tyr1068) mouse mAb (Cell Signaling, 2236), anti-V5 mouse mAb (Invitrogen, R960-25), and horseradish peroxidase (HRP)-conjugated Streptavidin (Pierce, 21130). Anti-mouse and anti-rabbit secondary antibodies conjugated to HRP were from Cell Signaling.

### Western blot analysis

Cells were washed 2 times in cold phosphate-buffered saline (PBS), then lysed in cell lysis buffer (50 mM HEPES, 200 mM NaCl, 25 mM NaF, 50 mM β-Glycerophosphate, 1 mM MgCl2, 1% Triton X-100, 1 mM DTT, 1 mM Na_3_VO_4_, 1 μg/mL aprotinin, and 1 μg/mL leupeptin). The lysates were centrifuged at 13,000 rpm for 15 minutes at 4°C, and equal amounts of protein were diluted with Laemmli sample buffer, boiled, and subjected to SDS-PAGE. The proteins were transferred onto PVDF membranes, and the membranes were blocked with 5% nonfat dry milk in TBST (20 mM Tris, 137 mM NaCl, pH 7.4, 0.05% Tween-20). The membranes were incubated with the primary antibodies either overnight at 4°C or for 1 hour at room temperature, then washed 3 times with TBST. Anti-mouse and anti-rabbit secondary antibodies were diluted in TBST and incubated with the membranes for 1 hour at room temperature. Membranes were washed 3 times in TBST, and visualized on x-ray film using Western Lightning Plus-ECL (PerkinElmer).

### Transamidation activity assay

15 μg of cell lysates were incubated in a buffer containing 10 mM DTT, 10 mM CaCl_2_, and 62.5 μM BPA for 15 minutes at room temperature. For analysis of the inhibition of cross-linking activity, cell lysates were either incubated with Z-Don for 30 minutes prior to the addition of the transamidation buffer, or incubated with 200 μM MDC. The reactions were quenched by the addition of Laemmli sample buffer, and boiled for 5 minutes. The proteins were separated by SDS-PAGE, transferred onto a PVDF membrane, and blocked overnight in BBST (100 mM boric acid, 20 mM sodium borate, 0.01% SDS, 0.15% Tween-20, 80 mM NaCl) containing 10% BSA. The membranes were then incubated in BBST containing 5% BSA and 1:5000 HRP-conjugated streptavidin for 1 hour at 4°C, washed thoroughly in BBST, and those proteins that incorporated BPA were visualized on x-ray film using Western Lightning Plus-ECL.

### Neurosphere forming assay

MES GSCs were dissociated into single cells, and seeded at 5 × 10^3^ cells/well in 12-well plates. Spheres were counted after 3 days on an inverted microscope. MES GSC aggregates containing > 4 cells were defined as neurospheres.

### Proliferation assay

MES GSCs were dissociated into single cells, and 12-well plates were seeded with 5 × 10^3^ cells/well. After 3 days, each well was supplemented with 2 mL GSC medium containing growth factors and inhibitors. Viable cells were quantified after 2, 4, and 6 days by staining with Trypan Blue Solution and counting cells on a hemocytometer. Those cells that excluded the dye were considered viable cells.

### Cell viability assay

For assays carried out in supplement-free medium, MES GSCs were seeded at 2 × 10^4^ cells/well in 12-well plates in either GSC medium or DMEM/F12, with or without inhibitors. 48 hours later, the cells were collected and stained with Trypan Blue Solution. Viable and dead cells were counted on a hemocytometer, and at least 200 cells were counted for each sample. For assays carried out in complete GSC medium with a combination of Z-Don and either TMZ or radiation, the cells were seeded at 5 × 103 cells/well in 12-well plates, and treated with IC_50_ doses for each treatment (MES GSC 13 cell line: 30 μM Z-Don, 1 μg/mL TMZ, 3 Gy; MES GSC 326 cell line: 20 μM Z-Don, 1 μg/mL TMZ, and 3 Gy). Radiation was carried out 2-4 hours after seeding the cells. Fresh medium containing growth factors and inhibitors was added to each well after 3 days. After 6 days, the viable and dead cells were counted as described above.

### RNA isolation and quantitative real-time PCR

Cells were washed 2 times in cold PBS, and RNA was isolated using Qiagen QIAshredder columns and the Qiagen RNeasy Mini Kit according to the manufacturer's instructions. The Superscript III First-Strand Synthesis System was used to synthesize cDNA from equal amounts of RNA according to the manufacturer's instructions. For quantitative Real-Time PCR (qRT-PCR), each master mix containing cDNA, primers (0.3125 μM each), water, and iTaq Universal SYBR Green Supermix was divided into triplicate reactions. qRT-PCR was carried out using the 7500 Fast Real-Time PCR System (Applied Biosystems) with the following amplification program: 50°C for 2 minutes, 95°C for 10 minutes, and 40 cycles of 95°C for 15 seconds followed by 60°C for 1 minute. Dissociation curves were carried out to analyze amplicon quality, and GAPDH was used as an internal control. Primer sequences used in this study are: ALDH1A3 forward: TGGATCAACTGCTACAACGC; ALDH1A3 reverse: CACTTCTGTGTATTCGGCCA; tTG forward: CTTTGTCTTTGCGGAGGTC; tTG reverse: CAGTTTGTTCAGGTGGTTCG; GAPDH forward: GAAGGTGAAGGTCGGAGTCA; GAPDH reverse: TTGAGGTCAATGAAGGGGTC.

### Cloning and DNA constructs

The EGFRvIII overexpression construct was previously described in [54]. The following shRNA constructs were purchased from Sigma: ALDH1A3 clone 1: TRCN0000027144; ALDH1A3 clone 2: TRCN0000027160. ALDH1A3 was amplified from cDNA isolated from MES GSC 1123 cells using the following primers: ALDH1A3 forward: CACCATGGCCACCGCTAACGG; ALDH1A3 reverse: GGGGTTCTTGTCGCCAAG. The PCR product was purified and ligated into the pcDNA3.1D/V5-His-TOPO vector (Invitrogen) according to the manufacturer's instructions. The V5-tagged ALDH1A3 was then cloned into a lentivirus overexpression construct (pCDH-CMV-MCS-EF1-Puro, System Biosciences). The ALDH1A3(C314A) lentivirus overexpression construct was generated using the following primers: ALDH1A3(C314A) forward: AAGGCCAGTGTGCCACGGCAGCCT; ALDH1A3(C314A) reverse: AGGCTGCCGTGGCACACTGGCCTT.

### Transfection, lentivirus generation, and transduction

The EGFRvIII overexpression construct was transfected into U87 cells with Lipofectamine according to the manufacturer's instructions. Viruses were generated by co-transfecting HEK293T cells with the lentiviral expression construct of interest and the packaging plasmids pMD-G and pCMV.d8.2 using PEI in DMEM containing 10% FBS. The medium was changed to serum-free DMEM the following day. The medium containing viral particles was collected after 24 hours and 48 hours, combined, centrifuged at 1800 rpm for 5 minutes, aliquoted, and stored at -80°C. Viral transduction was carried out by dissociating GSCs into single cells, and incubating them with viral particles and polybrene (10 μg/mL). 24 hours later, the cells were washed and incubated in fresh GSC medium, and selected with puromycin (MES GSCS: 4 μg/mL; PN GSCS: 0.5 μg/mL).

### FACS analysis

For cell sorting based on ALDH1 activity, cells were collected, dissociated into single cells, and counted on a hemocytometer. They were then incubated with or without the ALDH1 inhibitor DEAB (150 μM), and stained using the ALDEFLUOR Kit according to the manufacturer's protocol. The cells were then sorted on a BD FACSAria (BD Biosciences), and those cells in the top 15 percent and bottom 15 percent of ALDH1 activity were collected for RNA isolation and qRT-PCR, as described above.

### Statistical analysis

Each experiment was carried out a minimum of three times. For data presented as bar or line graphs, error bars represent the standard error of the mean (SEM). Statistical significance was calculated in Excel using F-tests for sample variance and Student's t tests for significance. *p* values < 0.05 were considered statistically significant, and were indicated with asterisks.

## SUPPLEMENTARY MATERIALS FIGURE


