# Supplementary material for: The stem cell/cancer stem cell marker ALDH1A3 regulates the expression of the survival factor tissue transglutaminase, in mesenchymal glioma stem cells
Source: Oncotarget. 2017 Mar 22;8(14):22325–43. doi: 10.18632/oncotarget.16479 (PMC5410226; doi:10.18632/oncotarget.16479)
Supplement: Supplementary file 1 [file oncotarget-08-22325-s001.pdf]

The stem cell/cancer stem cell marker ALDH1A3 regulates the expression of the survival factor tissue transglutaminase, in mesenchymal glioma stem cells

Supplementary Material

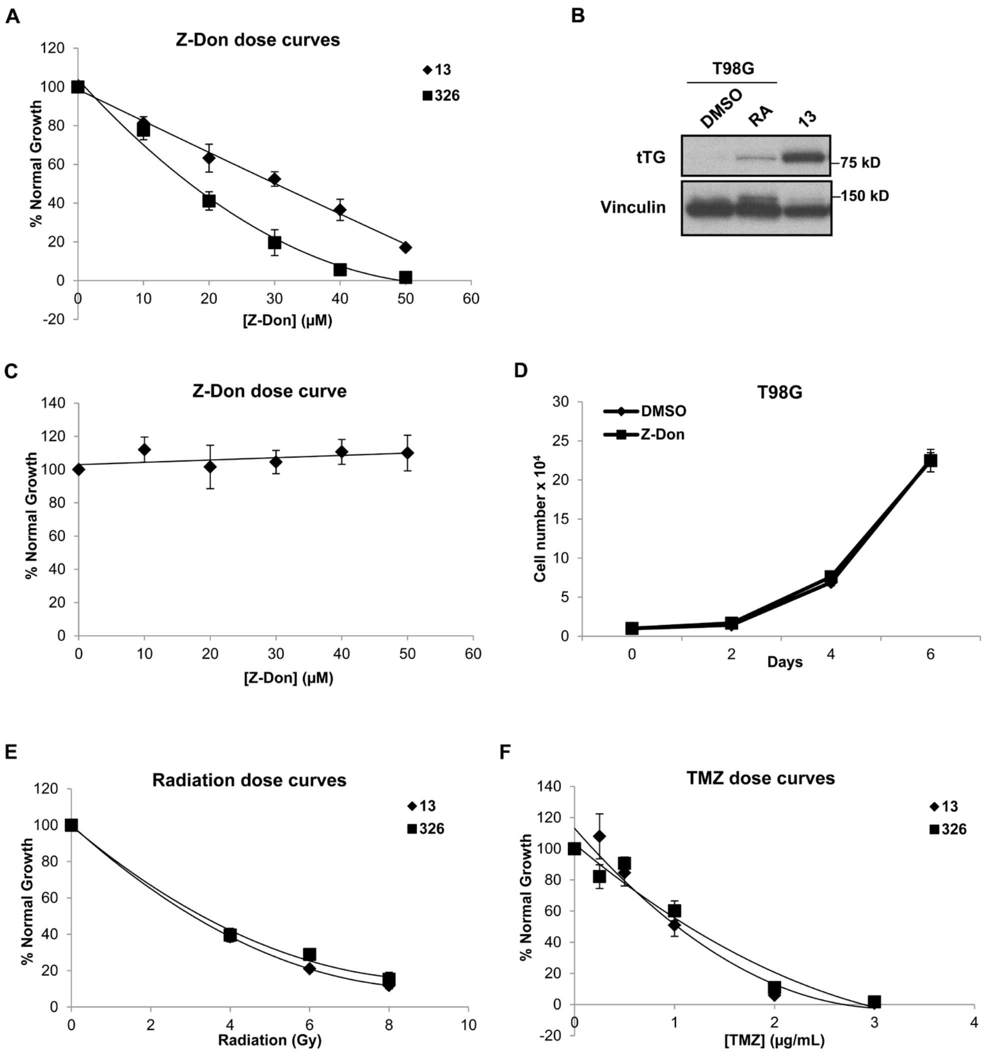

Supplemental Figure 1: Determination of dose curves and IC_{50} values for Z-Don, radiation, and temozolomide in MES GSCs. A. MES GSC 13 and 326 cells were dissociated into single cells, seeded at 5 x 10^3 cells/well in 12-well plates, and treated in triplicate with the indicated concentrations of Z-Don. Fresh medium was added 3 days later, and viable cells were counted after 6 days of growth. B. T98G glioblastoma cells were treated with or without 0.5 μM RA for 2 days. Whole cell lysates were collected, and immunoblotted in parallel with a whole cell lysate from MES GSC 13 cells using tTG and Vinculin antibodies. C, D. T98G cells were dissociated into single cells, and 12-well plates were seeded with 1 x 10^4 cells/well. 5 hours later, the medium was replaced, and the cells were treated in triplicate with either the indicated concentrations of Z-Don (C), or DMSO or 50 μM Z-Don (D). The medium was refreshed after 3 days. C. Viable cells were counted after 6 days of growth to determine a Z-Don dose curve. D. Viable cells were counted at the indicated time points to determine cell proliferation. E. MES GSC 13 and 326 cells were dissociated into single cells, and seeded at 5 x 10^3 cells/well in triplicate in 12-well plates. 4 hours later, the cells were exposed to the indicated levels of radiation. Fresh medium was added 3 days later, and viable cells were counted after 6 days of growth. F. MES GSC 13 and 326 cells were dissociated into single cells, seeded at 5 x 10^3 cells/well in 12-well plates, and treated in triplicate with the indicated concentrations of TMZ. Fresh medium was added 3 days later, and viable cells were counted after 6 days of growth.
